# Breast Cancer Chemoresistance: Insights into the Regulatory Role of lncRNA

**DOI:** 10.3390/ijms242115897

**Published:** 2023-11-02

**Authors:** Seyedeh Tayebeh Ahmadpour, Charlotte Orre, Priscila Silvana Bertevello, Delphine Mirebeau-Prunier, Jean-François Dumas, Valérie Desquiret-Dumas

**Affiliations:** 1 Nutrition, Croissance et Cancer, Inserm, UMR1069, Université de Tours, 37032 Tours, France; priscila.bertevello@univ-tours.fr (P.S.B.); jean-francois.dumas@univ-tours.fr (J.-F.D.); 2 Inserm U1083, UMR CNRS 6214, Angers University, 49933 Angers, France; charlotte.orre@univ-angers.fr (C.O.); deprunier@chu-angers.fr (D.M.-P.)

**Keywords:** breast cancer, chemoresistance, long noncoding RNA, metabolism

## Abstract

Long noncoding RNAs (lncRNAs) are a subclass of noncoding RNAs composed of more than 200 nucleotides without the ability to encode functional proteins. Given their involvement in critical cellular processes such as gene expression regulation, transcription, and translation, lncRNAs play a significant role in organism homeostasis. Breast cancer (BC) is the second most common cancer worldwide and evidence has shown a relationship between aberrant lncRNA expression and BC development. One of the main obstacles in BC control is multidrug chemoresistance, which is associated with the deregulation of multiple mechanisms such as efflux transporter activity, mitochondrial metabolism reprogramming, and epigenetic regulation as well as apoptosis and autophagy. Studies have shown the involvement of a large number of lncRNAs in the regulation of such pathways. However, the underlying mechanism is not clearly elucidated. In this review, we present the principal mechanisms associated with BC chemoresistance that can be directly or indirectly regulated by lncRNA, highlighting the importance of lncRNA in controlling BC chemoresistance. Understanding these mechanisms in deep detail may interest the clinical outcome of BC patients and could be used as therapeutic targets to overcome BC therapy resistance.

## 1. Introduction

Breast cancer (BC) is the second most common cancer worldwide and the most common cancer in women [1]. Immunohistochemical studies have divided BCs into three main types with different percentages and prognoses: (1) Estrogen receptor (ER+) and progesterone (PR+) positive, (2) Human epidermal growth factor receptor 2-positive (HER2+), and (3) Triple-negative (TNBC) BCs. Receptor-positive BC has the best prognosis, while TNBC, which is the most heterogeneous type of BC, has higher recurrence and shorter overall survival compared to the other two types [2]. Various cell populations are involved in breast tumor heterogeneity and respond differently to treatment. Among these, two key populations play vital roles: persister cells and cancer stem cells (CSCs). Persister cells are a subset of cancer cells that can survive initial treatment, often by entering a dormant state and play a critical role in drug resistance. These cells can contribute to treatment failure by later regenerating the tumor, causing recurrence [3]. Cancer Stem Cells (CSCs) are a subset of cells with self-renewal properties and the ability to give rise to more differentiated cancer cells [4]. We will address the topic of CSCs in Section 7.

Understanding the interplay between these cell populations and their roles in breast tumor heterogeneity is crucial for developing effective treatment strategies. Research aims to delineate the specific molecular pathways and factors that drive the persistence of persister cells and the regenerative capacity of CSCs. By targeting these populations, scientists and clinicians hope to improve treatment outcomes and reduce drug resistance. Chemoresistance, known as multidrug resistance (MDR), is a major obstacle against tumor control. Several mechanisms of BC drug resistance have been highlighted including aberrant lipid metabolism [5,6,7,8], increased drug efflux [9,10,11], insensitivity to death stimuli [12,13], cancer stem cells (CSC) regulation [4], and epigenetic modifications [14,15,16]. However, the underlying mechanisms provoking drug resistance are far from being deeply understood. In the upcoming section, we will provide an overview of key mechanisms in drug resistance.

## 2. Exploring Drug Resistance Mechanisms: An Overview of Key Insights

Chemoresistance, especially the development of MDR, is a significant challenge in cancer treatment. Several molecular mechanisms are being studied in relation to chemoresistance and the acquisition of the MDR phenotype. These mechanisms are complex and often interrelated, making the understanding and management of chemoresistance an ongoing research endeavor. Some of the key mechanisms and factors involved in chemoresistance and MDR are as follows:

- Tumor microenvironment: The tumor microenvironment, including interactions with stromal cells, immune cells, and extracellular matrix components, can promote drug resistance. Cancer-associated fibroblasts (CAFs) in the tumor microenvironment can promote chemoresistance by secreting factors that protect cancer cells from the effects of chemotherapy [17]. Another important factor in the tumor microenvironment is immunosuppressive cells. Tumor-associated macrophages (TAM) often contribute to the creation of an immunosuppressive microenvironment within the tumor [18]. TAMs can release various cytokines, growth factors, and chemokines that can protect cancer cells from the toxic effects of chemotherapy. They also release factors like interleukin-10 (IL-10) and transforming growth factor-beta (TGF-β), which suppress the immune response. This immunosuppression can lead to decreased recognition and elimination of cancer cells by the immune system, making them less responsive to chemotherapy. Interestingly, glucose metabolic reprogramming plays a crucial role within the tumor microenvironment, particularly in tumor-associated stromal cells. TAMs, based on their polarization, exhibit distinct metabolic profiles, with M2 macrophages showing a high capacity for glucose uptake, which contributes to chemoresistance and metastasis. Lactate produced by tumor cells also influences TAM polarization. Furthermore, CAFs shift from oxidative phosphorylation to aerobic glycolysis, promoting MDR in BC cells. This glucose metabolism reprogramming within stromal cells in the tumor microenvironment plays a significant role in tumor progression, metastasis, and therapy resistance [19].
- Tumor Heterogeneity: Intratumor heterogeneity, where different cells within a tumor have distinct genetic and epigenetic profiles, can lead to the survival of drug-resistant cell populations [20]. Tumor heterogeneity in BC results from a complex interplay of internal and external factors. Internally, genomic variations, phenotypes, and gene expression, including chromosomal rearrangements, contribute to intratumoral and patient heterogeneity. Externally, factors such as hypoxia render chemotherapeutic drugs less effective by altering metabolic enzyme expression and key drug targets. For instance, Hypoxia inducible factor-1 (HIF-1) regulates drug efflux pumps like P-glycoprotein (P-gp) and breast cancer resistance protein (BCRP) [21,22]. Interestingly, it has been suggested that the alteration in tumor oxygenation, and possibly the effects of hypoxia, is a critical factor influencing the response to chemotherapy and the development of chemoresistance in BC [23]. Understanding these influences is vital for improving BC treatment and combating resistance. Another factor that is involved in breast tumor heterogeneity is CSCs. CSCs are pivotal contributors to chemoresistance in BC. They exhibit elevated expression of various ABC transporters, including P-gp, ABCG2, and ABCC1. CSCs can also foster drug resistance through their proficient DNA repair mechanisms and the upregulation of anti-apoptotic factors. CSCs in breast cancer are characterized by specific markers that distinguish them from other tumor cells. These markers, such as CD44+ CD24− and ALDH1, enable the identification and isolation of CSCs. These cells exhibit higher tumorigenicity and resistance to therapy, contributing significantly to the heterogeneity of breast cancer [20].
- Signaling Pathways: Signaling pathways can contribute to chemoresistance in BC.
- Upstream Signaling Pathways: Upstream signaling pathways, such as EGFR, HER-2, and IGF-1R, can become activated in breast cancer cells. When these pathways are activated, they may promote cell survival and growth. This heightened activity can make cancer cells less responsive to chemotherapy. For example, EGFR and HER-2 signaling are associated with tamoxifen resistance [20,24].
- Downstream signaling pathways: Downstream signaling pathways, like the PI3K/AKT/mTOR and RAS/MAPK/ERK pathways, can be activated in response to upstream signaling. When these pathways are active, they may support cell survival and resistance to chemotherapy. For instance, the activation of the PI3K/AKT/mTOR pathway is linked to resistance to endocrine therapies, and this can interfere with the effects of chemotherapy [25].

These pathways can potentially interfere with the ability of chemotherapy drugs to induce DNA damage or trigger cell death [26]. The activation of these signaling pathways in BC cells can enhance their survival and growth, which can, in turn, make them less responsive to chemotherapy. These pathways may influence how cancer cells respond to DNA damage induced by chemotherapeutic drugs and, as a result, contribute to chemoresistance. Understanding these signaling pathways and their impact on drug resistance is vital for developing more effective cancer treatments.

- Immune Checkpoint Pathways: Immune checkpoint molecules, like PD-1 and CTLA-4, can be upregulated in cancer cells. When these molecules interact with their ligands on immune cells, they can inhibit the immune response against the tumor. This immunosuppressive interaction can contribute to resistance to immune-based therapies like immune checkpoint inhibitors, which are designed to activate the immune system against cancer cells. Chemotherapy has been shown to impact various immunosuppressive cell populations, which may influence breast cancer immunity and the efficacy of immunotherapeutic strategies. For instance, chemotherapy can reduce the abundance of Treg cells and switch the immune response from a silent state to an active one. Additionally, it can modulate the polarization of M2 macrophages to M1 macrophages, enhancing the antitumor immune response. Furthermore, chemotherapy can affect the expression of immune checkpoints like PD-L1 and inhibit tumor immunosuppression, thus potentially sensitizing tumors to immunotherapies. In a study of chemotherapy-treated tumors, it was observed that doxorubicin treatment alone did not significantly impact the immune landscape or lead to immune cell infiltration into the tumors. However, combining chemotherapy with immune checkpoint inhibitors targeting PD-L1 and CD80 improved treatment response, with some tumors completely eradicated. While these findings are promising, the complexity of the tumor microenvironment and the variations in immune checkpoint expression in BC pose challenges to fully understanding the impact of chemotherapy on immunity. Further research is needed to explore the multifaceted effects of chemotherapy on specific immune cell subtypes and immune checkpoint expression in BC [27,28].

Understanding these mechanisms is crucial for developing strategies to overcome chemoresistance and improve cancer treatment outcomes.

## 3. Long Noncoding RNAs

Long noncoding RNAs (lncRNAs) have been demonstrated to play prominent roles in many critical cellular processes, such as transcription, translation, stem cell differentiation, cell autophagy, apoptosis, and epigenetic control [29]. The lncRNAs are a subclass of noncoding RNAs that do not encode functional proteins. They are composed of more than 200 nucleotides and are usually divided into five categories according to their location relative to adjacent protein-coding genes, including bidirectional, antisense, intergenic, intronic, and sense lncRNAs [30]. LncRNAs exert biological functions by regulating the expression of multiple target genes in cellular processes and diseases by different mechanisms that can be classified into four main archetypes (Table 1). The guide function corresponds to the interaction of lncRNA with transcription factors or chromatin-modifying enzymes to activate the transcription of target genes. The scaffold function involves the role of lncRNA in stabilizing ribonucleoprotein complexes to regulate transcription. The decoy function of lncRNA enables their binding to regulatory protein to inhibit transcription or to miRNA to prevent their interaction with mRNA. Finally, the signaling function is involved in the inhibition of gene transcription, either directly or by interaction with chromatin-modifying enzymes [31,32,33,34,35]. In cancer, transcriptomic studies have highlighted the dysregulation of the expression of various lncRNAs in relation to the formation and the progression of the tumor [36]. Growing evidence suggests also the involvement of lncRNAs in developing BC chemoresistance [37,38,39,40,41,42,43,44,45,46,47,48,49,50,51,52]. For example, lncRNAs MALAT-1, HOTAIR, and H19 are proposed to have important associations with BC development and chemoresistance through alteration of mechanisms involved in apoptosis and efflux transporters activity [47,53,54]. Therefore, understanding such mechanisms that are deregulated by lncRNAs can improve our knowledge of potential target lncRNAs in BC and can provide new treatment strategies for controlling morbidity and mortality rates. In this review, we discuss the most studied mechanisms that are deregulated via lncRNAs involved in BC tumorigenesis with a special focus on drug chemoresistance.

## 4. Role of lncRNA in BC Chemoresistance through Regulation of Metabolism

It is well admitted that cancer cells endure profound metabolic adaptations, enhancing anabolic processes to sustain their proliferation [55]. Increasing evidence also demonstrates that metabolic flexibility is a crucial property enabling cancer cells to adapt their metabolism in response to the stress conditions triggered by chemotherapy [56]. These metabolic modifications concern both glycolytic and oxidative metabolism but also lipid and amino acid degradation and are involved in the development of chemoresistance [57].

### 4.1. Glucose Metabolism in Chemoresistant BC Cells

Consistent studies show that the energetic metabolism of BC-resistant cells relies mainly on glycolysis [58,59,60]. The study by Xu et al. [61], in 2018, identified FGFR4 (Fibroblast Growth Factor Receptor 4) as an important regulator of BC cell resistance to doxorubicin (DOX). In these cells, activation of FGFR4 enabled the phosphorylation of FGF receptor substrate 2, which, in turn, stimulated the ERK1/2 pathway. This activation resulted in an increase in the expression of glycolytic enzymes and accelerated the glycolytic flux. Later, it was shown in TNBC MDA-MB-231 and in MCF7 cells resistant to DOX that the small GTPase protein RAC1 (Ras-related C3 botulinum toxin substrate 1) was involved in the phosphorylation of MAPK/ERK pathways, which, in turn, stimulated the first steps of glycolysis and the non-oxidative phase of the pentose phosphate pathway [62]. The activity of hexokinase 2 (HK2), the glycolytic enzyme responsible for the conversion of glucose to glucose-6-phosphate, is also highly modified in BC cells resistant to tamoxifen and paclitaxel [63,64]. In the last model, HK2 activation was mediated by a direct interaction with PIM2 (Proviral Insertion in Murine Lymphomas 2), which phosphorylated and consequently activated HK2 [64]. Interestingly, PIM2 can also phosphorylate the glycolysis regulator PFKFB3 (6-phosphofructo-2-kinase/fructose 2,6-bisphosphatase 3), overexpressed in many cancer cells, leading to activation of PFK1 (phosphofructokinase 1), the third enzyme of the glycolytic pathway [65]. Finally, isoform 2 of pyruvate kinase (PKM2), predominantly expressed in cancer cells, was shown to form dimers with low catalytic activity [66], resulting in the accumulation of upstream glycolytic intermediates, which are derived from anabolic pathways [67].

### 4.2. Regulation of Glucose Metabolism by lncRNA in Chemoresistant BC Cells

**Figure 1:**
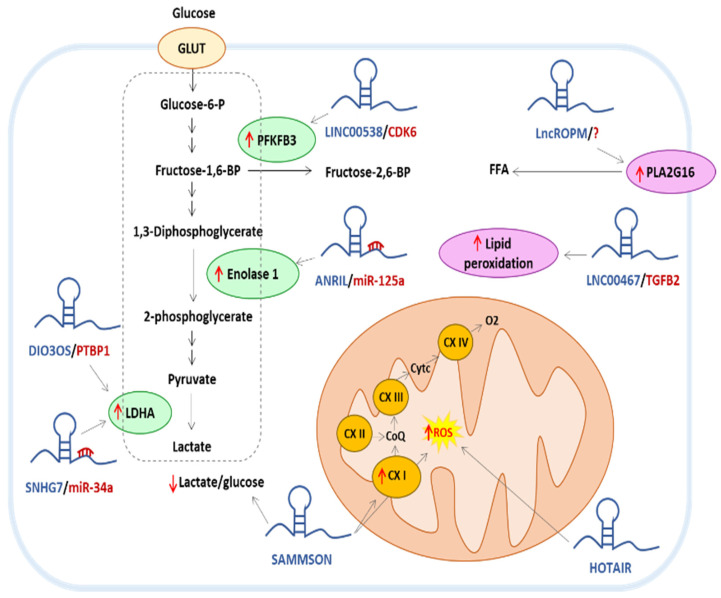
LncRNA players in regulation of metabolism associated with BC chemoresistance; LncRNAs control the activity of proteins and enzymes involved in glucose metabolism (green), lipid metabolism (purple), and mitochondrial metabolism (orange) regulating therapy response of BC. gray arrows show positive regulation by lncRNA. Red arrows show increased or decreased activity of the target enzyme.

Recent literature describes the role of lncRNA in the glycolytic rewiring of chemoresistant BC cells (Figure 1 and Table 2). For example, the lncRNA DIO3OS has been found overexpressed in aromatase-inhibitor-resistant breast tumors and its expression correlated with a poor prognosis in these patients. The mechanism of action of DIO3OS involved its interaction with polypyrimidine tract binding protein 1 (PTBP1), which stabilized the mRNA of lactate dehydrogenase A (LDHA), thereby upregulating LDHA expression and promoting glycolytic metabolism [68]. Moreover, it was found in TNBC cells that the lncRNA ANRIL was involved in chemoresistance to DOX by promoting glycolytic metabolism. The mechanism of action relied on the removal of the suppressive effect of miR-125a on the glycolytic enzyme enolase 1 [69]. The intergenic lncRNA LINC00538, also called YIYA, is also described as an important regulator of BC cell metabolism. By its interaction with CDK6 (cyclin-dependent kinase 6), YIYA promoted the phosphorylation and, consequently, the activation of the glycolysis enzyme PFKFB3 (fructose bisphosphatase PFK2) [70]. The lncRNAs from the Small Nucleolar RNA Host genes family (SNHG) are also involved in the regulation of glucose metabolism in BC cells. The most studied is SNHG3, a lncRNA located on chromosome 1, which has been found both in the nucleus and in the cytoplasm of various cell types. Interestingly, SNHG3 was also identified in the exosomes secreted from cancer-associated fibroblasts (CAF) that were captured by MDA-MB 453 BC cells. Once in the cytoplasm of these cells, SNHG3 was able to titrate the microRNA (miRNA) miR-330-5p. This interaction resulted in a lifting of the repression exerted by miR-330-5p on the glycolytic enzyme pyruvate kinase PKM2 [71]. The lncRNA SNHG7 was detected as overexpressed both in BC cells and tissues resistant to DOX and paclitaxel where it inhibited the expression of miR-34a, a negative regulator of LDHA expression [72]

Glycolysis enzymes are key targets of lncRNA. These molecules mainly stimulate glycolytic enzymes by repressing miRNA action and by promoting posttranslational modifications. Targeting these lncRNAs in BC-resistant cells enables the glycolytic switch to be alleviated, which is an important feature of chemoresistant cells.

### 4.3. Lipid Metabolism in Chemoresistant BC Cells

Aberrant lipid metabolism has been shown to trigger better survival of chemoresistant malignant cells and is associated with a poor prognosis [73,74]. However, underlying mechanisms are far from understood. In cancer cells, reprogramming of lipid metabolism can occur during both de novo biosynthesis and the catabolic pathway [75,76]. To support tumor growth and metastasis, some cancer cells use free fatty acids as a source of energy through the fatty acid oxidation (FAO) pathway [77]. An important enzyme involved in the de novo biosynthesis of fatty acids (FA) is FA synthase (FASN) [78]. Several studies show the involvement of FASN in BC chemoresistance. In BC cells resistant to anti-HER-2 therapies, increased FASN activity was related to HER2 overexpression, which occurs in approximately 15–30% of BC [5,6,7]. For example, in trastuzumab-resistant HER2-positive BC cells, overexpression of FASN has been observed [79]. Interestingly, the simultaneous blocking of FASN and HER2 pathways significantly increased the anti-tumor effect of anti-HER2 drugs for resistant BC cells [79]. FASN overexpression can also play an anti-apoptotic role in promoting cancer chemoresistance. Liu and colleagues have observed that FASN overexpression caused palmitate overproduction, supporting its anti-apoptosis role [80]. Additionally, FASN overexpression inhibited *TNF-α* expression, which, in turn, suppressed NF-κB as well as neutral sphingomyelinase to reduce ceramide production, suppress caspase 8 activation, and inhibit apoptosis [81]. Interestingly, FASN inhibitors induce apoptosis and sensitized BC cells to DOX [82]. Comparative studies between DOX-resistant BC cell lines and parental cell lines have demonstrated that changes in plasma membrane lipid composition modify multidrug resistance by altering drug uptake. DOX-resistant MCF-7 BC cells have higher concentrations of sphingomyelin and cholesterol than sensitive cells resulting in a stiffer and less permeable plasma membrane [8]. The hydrophobic interaction of DOX with the membranes of resistant cells could cause the drug to be trapped in the lipid bilayer. The amount of palmitate is also increased in the plasma membranes of resistant cells. FASN has been shown to be highly expressed in the drug-resistant BC cell line MCF7/AdVp3000, increasing palmitate content, which modulates the transverse mobility of membrane components and disrupts DOX uptake [80]. It is clear that disturbing DOX uptake leads to decreased DOX-induced toxicity and tumor cell apoptosis.

### 4.4. Regulation of Lipid Metabolism by lncRNA in Chemoresistant BC Cells

LncRNAs participate in lipid metabolism in different ways to affect tumor progression (Figure 1 and Table 3). A novel metabolic-related lncRNA, termed lncROPM (a regulator of phospholipid metabolism), is highly expressed in BC stem cells (BCSC) and contributed to BCSC chemoresistance to DOX, cisplatin, and tamoxifen. Mechanistically, lncROPMs increase phospholipase A2 (*PLA2G16*) expression, which, in consequence, promotes phospholipid metabolism and the production of free fatty acid, especially arachidonic acid in BCSC. An abundant amount of arachidonic acid activates PI3K/AKT, Wnt/β-catenin, and Hippo/YAP signaling pathways to contribute CSC features. Interestingly, a strategy using the combination of clinic therapeutic drugs such as DOX, cisplatin, or tamoxifen combined with Giripladib (an inhibitor of cytoplasmic phospholipase A2) could efficiently eliminate BCSC and tumorigenesis [83]. The lncRNA LINC00467 has also been suggested to play a role in the signaling pathways of peroxisomal lipid metabolism through miRNAs targeting transforming growth factor beta 2 (*TGFB2*), existing in the TGF-β signaling pathway. In fact, a positive correlation between the expression levels of TGFB2 and LINC00467 was observed in BC patients with a significantly lower overall survival rate. Interestingly, both TGFB2 and LINC00467 were located on the short arm of chromosome 1, and they were co-amplified in BC samples. In addition, it has been found that LINC00467 may also be involved in the regulation of lipid peroxide metabolism and epithelial–mesenchymal transition (EMT) and its relevant signaling pathways. This suggests that LINC00467 may affect the metabolic reprogramming of BC cells, thereby improving the antioxidant capacity of cells and promoting cell proliferation [84].

Recently, a study by Zhang et al. [85] reported the role of a potential lncRNA OLMALINC (Oligodendrocyte Maturation-Associated Long Noncoding RNA) in BC initiation and progression. This lncRNA was first evidenced as an enhancer of the stearoyl-coenzyme A desaturase gene (*SCD1*) [86]. This enzyme catalyzes the synthesis of monounsaturated fatty acids, and a high expression of *SCD1* has been associated with poor prognosis in BC patients [87]. The lncRNA OLMALINC acts in cis on the promoter of *SCD1* by DNA–DNA looping interaction and promotes the *SCD1* expression [86]. In BC cells, OLMALINC is activated by PTHrP (PTH-related protein) in an SREBP1 (Sterol regulatory element-binding protein 1) dependent mechanism [85]. This activation results in an increase in expression of the rate-limiting enzyme of fatty acid unsaturation pathway FASD2 (Fatty Acid Desaturase 2).

In summary, lipid metabolism could be modified in chemoresistant BC cells by de novo biosynthesis and fatty acid oxidation providing energy and cell components necessary to support tumor growth. The lncRNA involved in lipid metabolism mainly acts by modulating cellular pathways involved in BC cell proliferation and signaling. However, the precise role of lncRNA on chemoresistance and its precise mechanisms need further investigation.

### 4.5. Mitochondrial Metabolism in Chemoresitant BC Cells

Mitochondria play an important role in energetic metabolism but also participate in the regulation of the reductive ratio (NAD+/NADH), reactive oxygen species (ROS), and calcium levels, all essential elements of cell homeostasis. As tumor cells undergo profound changes in metabolism, substrate supplies to the respiratory chain are thus modified, and consequently mitochondrial ATP production could be also affected. An increasing number of publications place the reprogramming of mitochondrial metabolism in BC cells as a consequence of the rewiring of glucose and fatty acid fluxes, and also directly implicate mitochondrial metabolism changes in the tumoral progression and chemoresistance [88,89].

Mitochondria are indeed at the crossroads of many metabolic pathways and play a key role in the metabolic flexibility required for cancer cells to adapt their metabolism to the stress conditions induced by chemotherapies and, therefore, are crucial players in resistance to anticancer drugs. Numerous publications report changes in mitochondrial metabolism and respiratory chain functioning in cancer cells resistant to chemotherapy but these adaptations are heterogeneous regarding the cell model and the chemotherapeutic agent. Notably, a subpopulation of stem cells, called tumor-initiating cells, has been identified as being involved in chemoresistance [90]. Interestingly, these cells display a specific energetic metabolism relying mainly on mitochondrial energy production at the expense of an increase in ROS production and an increase in mtDNA content, two conditions that render the cells prone to accumulate mitochondrial DNA somatic mutations, which can, in turn, accentuate mitochondrial dysfunction [91]. These results are still debated as another study reports on the contrary a lower content of mitochondria in murine BC tumor-initiating cells than in non-tumorigenic BC cells [92], and the signaling pathways involved in the regulation of mitochondrial transcription upon chemotherapy remain to be determined. The mitochondrial metabolism is tightly regulated by the mitochondrial dynamic, and an over-fragmented mitochondrial network has been evidenced in tamoxifen-resistant BC cells correlated with phosphorylation of the fission protein DRP1. Consequently, in these cells, respiratory chain complex assembly was impaired and glycolytic metabolism was increased, a metabolic reprogramming that increased tamoxifen chemoresistance [93]. Chang et al. also demonstrated that inhibition of mitochondrial fission by Mdivi-1 (Mitochondrial Division Inhibitor 1) combined with the pro-fusion cell-permeant peptide Pep1 improved the BC cells’ sensitivity to DOX in mice [94]. Conversely, it was demonstrated by Han et al. that mitochondrial fission rather improves the sensitivity of triple-negative MDA-MB 231 BC cells to cisplatine [95]. In chemoresistant BC cells, the structure and function of the respiratory chain are also deeply modified. Some studies evidenced an impairment of respiratory chain function with a decrease in OXPHOS and a misassembly of complex I in DOX-resistant BC cells [58], while recent studies reported that the metabolism of anthracycline-resistant BC cells mainly relied on OXPHOS [96,97]. Other studies claimed that the main metabolic characteristic of chemoresistant BC cells is their ability to switch between glycolysis and OXPHOS and to remodulate their mitochondrial structure to adapt to environmental stress and energetic demand [60,98]. Altogether, these results underline the heterogeneity in BC cells’ mitochondrial adaptations to chemotherapy and suggest that the molecular actors involved in these adaptations differed between cells.

### 4.6. Regulation of Mitochondrial Metabolism by lncRNA in Chemoresistant BC Cells

LncRNAs emerge as important regulators of mitochondrial functioning in BC cells since they can interact with the different actors of the energetic homeostasis described above (Figure 1 and Table 4).

Mitochondrial dynamics: The lncRNA MTFR2 (Mitochondrial Fission Regulator 2) has been shown in BC samples to increase invasion and tumor progression by modulation of metabolic orientation (glycolysis versus OXPHOS) by a Hypoxia Inducible Factor (HIF)-1alpha-dependent mechanism [99]. Similarly, the lncRNA RACGAP1P (pseudogene of Rac GTPase activating protein 1) was found overexpressed in BC tissues and its expression was associated with a poor prognosis. This lncRNA promoted mitochondrial fission by interacting with the miR-345-5p/RACGAP1 axis [100]. The study by Tian et al., in 2019, demonstrated that the overexpression of the lncRNA MPRL can increase the fission events and participate in cisplatin chemoresistance in tongue squamous cell carcinoma [101]. Whether such regulation is involved in BC chemoresistance is not yet elucidated and requires further investigation in BC research.

Mitochondrial DNA replication, transcription, and translation: We recently demonstrated that the intergenic lncRNA SAMMSON was involved in metabolic rewiring toward glycolysis and resistance to DOX in MCF-7 BC cells [102]. Moreover, the tumor suppressor lncRNA GAS5 could increase the translation of mitochondrial proteins and stimulate respiratory chain activity [103]. However, these results remain controversial since another study describes a pro-apoptotic role of GAS5 mainly by disruption of mitochondrial membrane potential and decrease in respiration [104]. Mitochondrial DNA can also produce lncRNA, either complementary to mitochondrial genes (lncND5, lncND6, and lncCytb) or as chimeric mitochondrial DNA such as sense mitochondrial ncRNA (SncmtRNA), identified in proliferating human tumor cells, and compounds of the mitochondrial 16S rRNA associated with an 815 nucleotide 5′-leader fragment deriving from the complementary strand. These lncRNAs are important in the regulation of mitochondrial DNA transcription but also serve as signaling molecules in retrograde signaling to the nucleus to coordinate mitochondrial and nuclear transcription [105].

Mitochondrial respiratory chain function: HOTAIR is one of the most studied oncogenic lncRNA and its expression is dysregulated in numerous cancer models. Recently, Li et al. demonstrated the role of this lncRNA in the resistance of MCF7 cells to DOX by a Pi3K/AKT/MTOR-dependent pathway [106,107]. Moreover, a quantitative proteomics analysis reveals that inhibition of HOTAIR results in a marked decrease in complex III UQCRQ subunit expression and induces a significant increase in ROS production [106]. In bladder cancer, the lncRNA UCA1 has been shown to increase mitochondrial respiratory chain activity and improve cell viability by interacting with miRNA miR-195 [108].

**Table 4:** Summary of studies that reported the role of lncRNAs in regulation of BC tumorigenesis and chemoresistance by modulating mitochondrial metabolism. ↑ shows positive regulation of target protein or miRNA.

LncRNA	Expression Pattern	Human/Animal/ Cell Lines	Treatment	Possible Pathway		Reference
SAMMSON	Up	MCF-7 cells resistant to DOX	Doxorubicin	↑ glycolytic pathway Complex I activity	Mitochondrial metabolism	[102]
HOTAIR	Up	MCF-7 cells	Doxorubicin	PI3K/AKT/mTOR UQCRQ expression	Mitochondrial metabolism	[106,107]
MTFR2	Up	Hs578T, MDA-MB-231 and MCF-7 cells	No	Modulation of metabolic orientation by HIF1a	Mitochondrial metabolism	[99]
RACGAP1P	Up	BC tissues	No	↑ in mitochondrial fission	Mitochondrial Fission	[100]

In contrast to glucose and lipid metabolism, only a few studies have described the role of lncRNA in mitochondrial metabolism, and even fewer in chemoresistance. Yet, mitochondria, by their central role in energetic production, are an important player in the metabolic reprogramming observed in BC cells and are also a target for some chemotherapeutic agents such as doxorubicin [109,110]. Identifying the lncRNAs that are involved in the regulation of mitochondrial metabolism could help to prevent the mitochondrial plasticity necessary for BC-resistant cells to adapt to cellular stress induced by chemotherapeutic agents.

## 5. Role of lncRNA in BC Chemoresistance through Regulation of Efflux Transporters

### 5.1. Efflux Transporters in Chemoresistant BC Cells

Efflux pumps are specialized proteins found in the cell membranes of various organisms, including bacteria, fungi, and human cells [111,112,113]. These pumps play a crucial role in the transport of various molecules, including toxins, drugs, and other substances, out of the cell. In human cells, efflux pumps are implicated in multidrug resistance, especially in cancer cells. They can pump out chemotherapy drugs, reducing their effectiveness and leading to treatment failure. This is a significant challenge in cancer therapy and it is imperative to conduct a detailed study of its functionality. To assess the functionality of efflux pumps in a cell or tissue sample, researchers can expose the cells to Hoechst dye with or without efflux pump inhibitors [114]. If the cells have active efflux pumps, they will pump out the dye, resulting in reduced intracellular Hoechst staining, and weaker fluorescence. This assay helps scientists evaluate the role of efflux pumps in drug resistance, particularly in cancer cells, and can be used to screen for potential inhibitors that may enhance the effectiveness of chemotherapy by blocking the efflux of drugs from cancer cells. Enhanced drug efflux in BC chemoresistance is mainly regulated by the ATP binding cassette (ABC) superfamily [115]. A number of ABC transporters are strongly implicated in the chemoresistance of numerous solid tumors, including BC [9]. In MCF-7 and MDA-MB-231 BC cells, the upregulation of ABC transporters appears to be a primary and rapid survival strategy within the process of cell-adhesion-mediated drug resistance (CAM-DR). Baltes and co-workers have observed that in MCF-7 and MDA-MB-231 BC cells, the sensitivity towards cisplatin, DOX, and mitoxantrone was decreased upon binding to collagen type 1 (COL1) or fibronectin. The intracellular concentrations of DOX and mitoxantrone were decreased upon cell cultivation on COL1 in relation to ABC efflux transporter activities. Interestingly, an ABC transporter inhibitor was able to re-sensitize COL1-treated cells to DOX and mitoxantrone treatment. Also, the study shows a link between ITGB1 and COL1. *ITGB1* is highly expressed in BC cells and plays a crucial role in mediating cell adhesion to COL1. In addition, the knockdown of *ITGB1* leads to a significant upregulation of three members of the ABC transporter, BC resistance protein (BCRP), P-glycoprotein 1 (P-gp), and Multidrug-resistant protein-1 (MRP1). This study suggests that BC cells cultured on ECM, particularly COL1, exhibit increased chemoresistance due to the upregulation of ITGB1, which, in turn, leads to the increased expression and activity of ABC transporters, resulting in higher drug efflux and reduced intracellular drug levels [10]. Fultang and colleagues have characterized ROR1 (Receptor Orphan tyrosine kinase-like Receptor 1) as an upstream regulator of ABCB1, a subfamily of ABC transporters. ROR1 regulates ABCB1 stability and transcription via MAPK/ERK and p53. They have also observed that ROR1 is overexpressed in BC chemoresistant cells MDA-MB-231 and SUM-159PT and correlates with poor therapy response and tumor recurrence. ROR1 inhibition was able to sensitize BC cells to DOX and cisplatin treatment [11]. *ABCC1/MRP1*, *ABCG2/BCRP*, and multidrug-resistant protein-8 (*ABCC11/MRP8*) were shown to be expressed significantly more and more frequently in TNBC and are associated with BC chemoresistance [116,117,118,119,120,121]. Hu and colleagues have investigated the role of TRPS1 (Transcriptional Repressor GATA Binding 1) in the acquisition of BC chemoresistance. Their findings show that TRPS1 regulated BCRP expression and efflux transportation. Overexpression of TRPS1 led to upregulation of BCRP while its inhibition resulted in repression of BCRP. Overall, high expression of TRPS1 confers MDR of BC, which is mediated by BCRP [122]. It has also been suggested that the NFkBP65 transcription factor modulates the resistance of MCF-7 BC to DOX through the ABC transporter, and suppression of its pathway was able to reduce the ABC transporter function and increase the intrinsic apoptotic pathway [123]. Interestingly, it was indicated that the combination treatment of curcumin and DOX enhanced the effects of DOX in DOX-resistant MCF-7 and MDA-MB-231 BC cells via the inhibition of the efflux function of ABCB4 [124]. Therefore, targeting efflux transporters in combination with other anti-cancer treatments could provide a promising strategy.

### 5.2. Regulation of Efflux Transporters by lncRNA in Chemoresistant BC Cells

**Figure 2:**
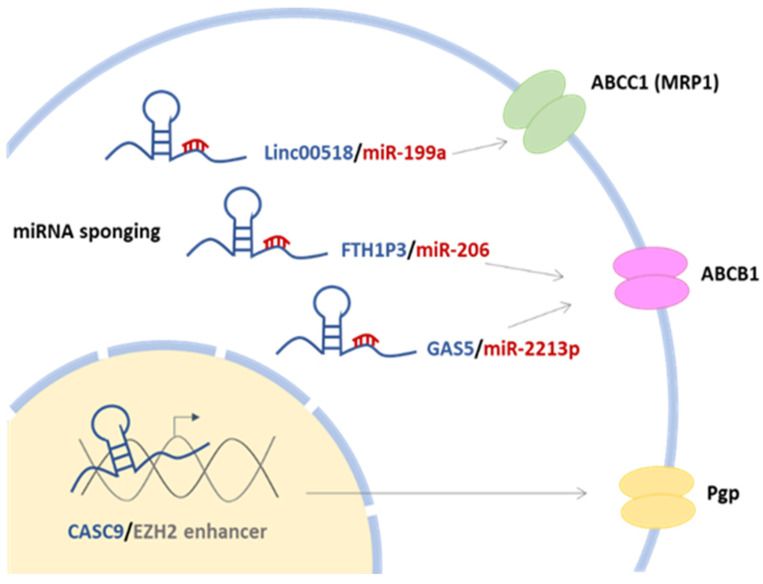
LncRNA players in regulation of efflux transporter associated with BC chemoresistance. LncRNAs can control the expression and activity of efflux pumps proteins through both transcriptional modification (CASC9 lncRNA) and miRNA sponging (Linc00518, FTH1P3, and GAS5 lncRNA).

One of the main regulatory aspects involved in ABC transporters-mediated chemoresistance is the interaction of lncRNAs and miRNAs (Figure 2 and Table 5).

Most lncRNAs are upregulated in chemoresistant cancers induced by overexpression of ABC transporters while miRNAs are downregulated. For instance, the upregulation of lncRNA HOTAIR in HCC is associated with an increased expression of the ABCB1 efflux transporter by sponging miR-145 [125]. LncRNAs are one of the main regulators of ABC efflux transporter’s activity and expression in BC-MDR. In adriamycin (ADR)-resistant BC tissue, lncRNA linc00518 regulates the expression of ABCC1 via sponging the miR-199a [40]. In this line, decreased expression of miR199a was associated with BC carcinogenesis and an increased level of miR-199a could reverse this process in vivo [126,127]. LncRNAs can also lead to chemoresistance by regulating the expression of P-gp (P-glycoprotein) in cancer cells [128,129,130]. For example, in MCF-7 DOX-resistant BC cells, lncRNA cancer susceptibility candidate 9 (CASC9) binds to enhancer of zeste homolog 2 (EZH2) to promote its expression, resulting in P-gp upregulation [130]. Wang and colleagues investigated the physiopathological role of lncRNA ferritin heavy chain 1 pseudogene 3 (FTH1P3) on paclitaxel (PTX) resistance in BC. They showed that lncRNA FTH1P3 was upregulated in paclitaxel-resistant BC tissue and cells (MCF-7/PTX and MDA-MB-231/PTX cells) compared to paclitaxel-sensitive tissue and parental cell lines (MCF-7, MDA-MB231). Silencing of FTH1P3 decreased the 50% inhibitory concentration (IC50) of paclitaxel and induced cell cycle arrest at the G2/M phase, while FTH1P3 overexpression showed the opposite effects. A study in vivo showed that the silencing of FTH1P3 suppressed the tumor growth of paclitaxel-resistant BC cells and ABCB1 protein expression in a xenograft mice assay. Mechanistically, FTH1P3 promoted ABCB1 protein expression through targeting miR-206, acting as a miRNA “sponge” [131]. LncRNAs seem to play a dual role in regulating cancer chemoresistance induced by efflux transporters. Opposite to previous studies, lncRNA GAS5 has been found to have an inhibitory role on efflux pumps in MCF-7/ADR BC cell lines. In ADR-resistant BC tissues and cells, the GAS5 level was downregulated compared to MCF-7 cells, whereas the ABCB1 level was upregulated. Interestingly, GAS5 overexpression significantly enhanced the ADR sensitivity to ADR treatment and inhibited the efflux function and expression of *ABCB1* in vitro, while its knockdown showed the opposite effects. Mechanistically, GAS5 binds to miR-221-3p and represses activation of its target DKK2, regulating *ABCB1* expression and ADR chemoresistance [129]. Thus, understanding the mechanisms through which lncRNAs regulate efflux transporter is crucial and could provide a new therapeutic target for controlling BC chemoresistance.

In summary, lncRNAs play complex and context-dependent roles in regulating ABC transporters, which are implicated in chemoresistance in various cancer types. Understanding these regulatory mechanisms can lead to the development of novel therapeutic targets to overcome chemoresistance and improve cancer treatment outcomes.

**Table 5:** Summary of studies that reported the role of lncRNAs in regulation of BC chemoresistance by modulating efflux transporters. ↑ shows positive regulation and ↓ show negative regulation of target protein or miRNA.

LncRNA	Expression Pattern	Human/Animal/ Cell Lines	Treatment	Possible Pathway	Dysregulated Mechanism	Reference
LINC00518	Up	MCF-7 parental and Adr-resistant	Adriamycin, Vincristine, Paclitaxel	↓ miR-199a/↑ ABCC1 (MRP1)	Efflux transporters	[40]
CASC9	Up	MCF-7 cells resistant to DOX, MDA-MB-231, MDA-MB-157, MDA-MB-468	Doxorubicin	↑ EZH2/P-gp	Efflux transporters	[130]
FTH1P3	Up	MCF-7 and MDA-MB-231 cells resistant to paclitaxel, BC human tissues (*n* = 15), Xenograft mice assay in vivo	Paclitaxel	↓ miR-206/↑ ABCB1	Efflux transporters	[131]
GAS5	Down	MCF-7 resistant to adriamycin, HEK293A, MDA-MB-231, and MDA-MB-468 cells	Adriamycin	↓ miR-2213p/DKK2/↑ *ABCB1*	Efflux transporters	[129]

## 6. Role of lncRNA in BC Chemoresistance through Regulation of Apoptosis

### 6.1. Inhibition of Apoptosis in Chemoresistant BC Cells

Apoptosis is an intrinsic programmed cell death (PCD) recognized as a positive strategy to induce the death of cancer cells [132]. PCD is controlled by a variety of Bcl-2-related genes that either promote or inhibit apoptosis. Bcl-2, Bcl-XL, Mcl-1, A1/BFL-1, and Bcl-W are antiapoptotic proteins, while Bax, Bcl-Xs, Bcl-xL/Bcl-2-associated death promoter, Bak, Bik, and Bid are proapoptotic [133]. The induction of PCD is often determined by the ratio of proapoptotic to antiapoptotic proteins. Malignant cells are able to dysregulate apoptotic machinery to evade cell death and facilitate carcinogenesis and chemoresistance. In TNBC, evasion of apoptosis was linked to chemoresistance to paclitaxel, DOX, and cyclophosphamide [12,13]. The disturbed balance between proapoptotic and pro-survival factors is one of the causes of apoptosis malfunction. Increased protein expression of pro-survival factors, such as Bcl-2 and Mcl-1, has been observed in altered apoptosis in BC cells and seems to be associated with intrinsic BC chemoresistance [134,135,136,137,138,139]. An interesting therapeutic approach is to inhibit these anti-apoptotic proteins using agents that mimic the Bcl-2 homology 3 (BH3) domains of the pro-apoptotic members of the Bcl-2 family, thus allowing the neutralization of these proteins [140]. ABT-737 is a BH3 mimetic molecule able to bind with high affinity to Bcl-2, Bcl-xL, and Bcl-w but fails to bind to Mcl-1. Resistance to ABT-737 was linked to increased Mcl-1 expression and, in many cases, this resistance was overcome by treatment with agents that downregulate, destabilize, or inactivate Mcl-1 [141]. It has been observed that ABT-737 can also sensitize BC cells overexpressing the BH3 protein [142]. Promotion of apoptosis can also be mediated by P53 activation. P53 induces apoptosis through regulation of both proapoptotic and antiapoptotic proteins [143]. Reports showed that Mouse double minute 2 (MDM2), a positive regulator of ERα transcriptional activity, can inhibit P53 activity and avoid apoptosis activation [144]. Moreover, MDM2 was found to be overexpressed in MCF7 cells, leading to increased rates of cell proliferation [145,146]. Therefore, it is probable that MDM2 is linked to BC chemoresistance through alteration of P53-induced apoptosis. The PI3K/Akt pathway is often activated in HER2+ tumors [147]. Some proapoptotic proteins like Bcl-xL and Bcl-2-associated death promoters can also be altered by activation of Akt, leading to NF-kB activation, thus resulting in transcription of pro-survival genes and decreased treatment efficiency [148,149]. Further studies are fundamental to deeply understand the mechanisms that regulate the alteration of apoptosis in drug chemoresistance.

### 6.2. Regulation of Apoptosis by lncRNA in Chemoresistant BC Cells

**Figure 3:**
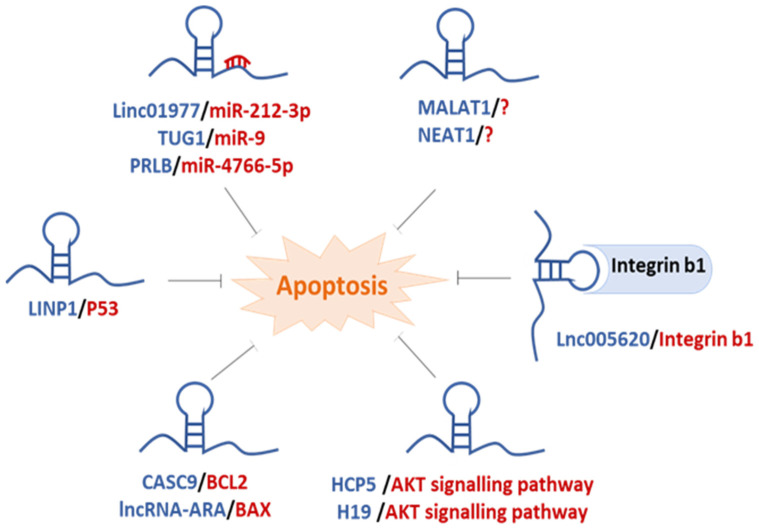
LncRNA players in regulation of apoptosis associated with BC chemoresistance. LncRNAs control apoptosis through several mechanisms such as miRNA sponging, P53 pathway, Bcl-2 family protein regulation, AKT pathway, and Integrin b1 regulation as well as stemness regulation. The interactive partner of MALAT1 and NEAT1 are not known and are shown by question mark in figure.

Recent research has implicated the involvement of lncRNA as regulators of apoptosis and BC chemoresistance through several mechanisms described below (Figure 3 and Table 6).

Regulation of miRNA: A number of findings suggest a strong correlation between dysregulated apoptosis and BC chemoresistance that are mediated through the lncRNA/microRNA axis. In most of these studies, lncRNA acts as a sponge and represses its microRNA target, which directly and/or indirectly inhibits and/or activates regulators of apoptosis, leading to chemoresistance [150,151,152]. For instance, in human BC tissues and MDA-MB-231 cells, increased expression of lncRNA-PRLB (progression-associated lncRNA in BC) was positively correlated with the extent of metastasis and shorter survival time of BC patients. Inactivation of miRNA miR-4766-5p by lncRNA-PRLB remarkably enhanced cell growth, metastasis, and chemoresistance [153]. Moreover, Li et al. have shown that higher expression of lncRNA LINC01977 was correlated with cancer progression and DOX chemoresistance of MCF-7 and MDA-MB-231 cells and inhibiting DOX-induced cell apoptosis via sponging of miR-212-3p [154]. Wang and colleagues have also shown a high expression of lncRNA TUG1 in BC tissues and a positive association with DOX resistance in BC cell lines. TUG1 downregulation was able to reverse DOX resistance in MCF-7/ADR cells. Mechanistically, TUG1 overexpression by sponging miR-9 and regulation of its downstream target eIF5A2 could modulate the sensitivity of BC cells to DOX [155].

P53 pathway: P53 is reported as a regulator of chemotherapy and radiotherapy resistance in MDR cancer cells [156]. It can upregulate the expression of proapoptotic proteins such as Puma, Noxa, Bid, and Bax and can also neutralize the anti-apoptotic activity of Bcl-2 and Bcl-xL [157]. Interestingly, p53 is identified as a regulator of several lncRNAs such as LINP1, MALAT1, GUARDIN, and PICART1. LINP1 overexpression could restore the metastatic effects of p53 in MDA-MB-231 and MDA-MB-468 cells. LINP1 enrichment played a critical functional role in chemoresistance by inhibiting chemotherapeutic-induced apoptosis of cells while its knockdown had the opposite effect [158]. MALAT1 binding to P53 and GUARDIN induction by P53 reduced P53-induced apoptosis while regulation of PICART1 lncRNA by P53 induced BC apoptosis [159,160,161].

Bcl-2 family proteins: Several lncRNAs were shown to be associated with altered apoptosis either through inhibition of proapoptotic proteins or activation of pro-survival proteins, linked to BC chemoresistance. LINC00628 lncRNA is characterized as a tumor suppressor in BC and can regulate apoptosis via targeting the BCL-2/BAX/Caspase-3 signaling pathway [162]. In BC tissues of patients, HOTAIR lncRNA has been reported to promote BC development and migration through upregulation of BCL-W (an anti-apoptotic protein) via miR-206 sequestering [54]. In addition, lncRNA CASC9 was found to promote DOX resistance of MCF-7 BC cells. Following CASC9 knockdown, the expression levels of Bcl-2, pro-caspase-3, and pro-caspase-9 decreased significantly while Bax, cleaved-caspase-3, and cleaved-caspase-9 increased markedly in MCF-7dox cells [130]. Jiang et al. reported the differential expression of LncRNA-ADR resistance-associated (lncRNA-ARA) in ADR-resistant MCF-7/ADR BC cells compared to parental MCF-7 cells. The authors showed that the depletion of lncRNA-ARA by ARA siRNA sensitized MCF-7/ADR cells to ADR. Also, they suggested that ARA plays a role in apoptosis and cell death as ARA depletion resulted in the upregulation of the pro-apoptotic Bcl-2-Associated X (BAX) protein and the downregulation of the anti-apoptotic B-cell lymphoma extra-large (Bcl-xL) [163].

AKT pathway: In clinical BC samples and cell lines, phosphatase and tensin homolog (PTEN) and PTEN pseudogene 1 (PTENP1) are concomitantly downregulated and the overexpression of PTENP1 and PTEN suppress BC progression. PTENP1 lncRNA activates the phosphatidylinositol-3 kinase (PI3K)/AKT pathway via the miR-20a/PTEN axis, leading to the proliferation, invasion, and drug resistance of BC cells, thus inhibiting apoptosis. Interestingly, PI3K inhibitor LY294002 or siAkt could prevent BC cell progression. The upregulation of PTENP1 significantly enhanced the ability of chemotherapy-induced apoptosis by increasing the levels of cleaved caspase3 and cleaved PARP1 in BC cell lines [164]. In line with this study, another study by Wu et al. found that upregulation of HCP5 lncRNA induces cisplatine chemoresistance of TNBC cells by inhibiting the expression of PTEN and promoting the level of p-Akt [165]. Moreover, it was shown that the knockdown of lncRNA H19 restores chemosensitivity in paclitaxel-resistant TNBC cells through triggering apoptosis and regulating the Akt signaling pathway. In H19 knock-out cells, caspase-3 expression was significantly increased, while the levels of pAkt and Bcl-2 expression were significantly decreased indicating a role for H19 lncRNA in regulating the Akt-mediated apoptotic signaling pathway [166].

Regulation of Integrin b1: In TNBC cells and human tissues, a high expression of the lncRNA lnc005620 was found. Integrin b1, encoded by the *ITGB1* gene, was identified as a downstream target of lnc005620. *ITGB1* was upregulated in TNBC patients who did not respond to epirubicin treatment compared to those who showed a response to epirubicin therapy. The silencing of *ITGB1* could promote apoptosis and inhibit the migration and invasion of epirubicin-resistant MDA-MB-231 cells [167].

Stemness regulation: Another mechanism to control apoptosis in cancer cells is through the regulation of stemness. Shin and co-workers found significantly higher expression of Nuclear-enriched abundant transcript 1 (NEAT1) in TNBC cells. The downregulation of NEAT1 sensitizes cells to chemotherapy, reduces the stemness, and increases apoptosis [168].

In summary, it appears that many lncRNA can contribute to BC chemoresistance through modulating apoptosis. Thus, the downexpression of CASC9, HCP5, H19, HOTAIR, LINC01977, LINP1, lnc005620, lncRNA-ARA, MALAT1, NEAT1, PRLB, and TUG1 significantly enhance the ability of chemotherapy-induced apoptosis in BC cell lines. On the contrary, the overexpression of LINC00628 and PTENP1 promotes cell apoptosis and sensitizes cells to chemotherapy. Concerning the main key ways in which lncRNA can exert its function and regulatory activities, it seems that HOTAIR, LINC01977, PTENP1, PRLB, and TUG1 act as an endogenous sponge of miRNA. Concerning MA-LAT1 and LINP1, they act on apoptosis by regulating the activity of p53. Finally, CASC9, HOTAIR, HCP5, H19, LINC00628, lnc005620, lncRNA-ARA, and PTENP1 act by inhibiting or promoting the expression of key regulators such as Bcl-2 family proteins and *ITGB1* or by inhibiting or activating key regulators such as the Akt pathway. Of note, some lncRNA can act on apoptosis via several of the above-mentioned key ways as, for example, for HOTAIR or PTENP1.

**Table 6:** Summary of studies that reported the role of lncRNAs in regulation of BC tumorigenesis and chemoresistance by modulating apoptosis. ↑ shows positive regulation and ↓ show negative regulation of target protein or miRNA.

LncRNA	Expression Pattern	Human/Animal/ Cell Lines	Treatment	Possible Pathway	Dysregulated Mechanism	Reference
LINC01977	Up	MCF-7 and MDA-MB-231	Doxorubicin	↓ miR-212-3p/↑ GOLM1	Apoptosis	[154]
MALAT1	Up	Human BC tissues/MCF-7 cells	Taxanes, Adriamycin	-	Apoptosis	[53]
TUG1	Up	MCF-7/ADR cells	Doxorubicin	↓ miR-9/↑ eIF5A2	Apoptosis	[155]
Lnc005620	Up	Human BC tissues, MDA-MB-231 cells	Epirubicin	↑ Integrin b1	Apoptosis	[167]
LINP1	Up	MDA-MB-231 and MDA-MB-468 cells	Doxorubicin, 5-fluorouracil	↑ P53	Apoptosis	[158]
NEAT1	Up	BC patients (*n* = 179) and normal controls (*n* = 192), In vivo xenograft animal model, MDA-MB-231 cells	Cisplatin, Taxol	-	Apoptosis/cancer stemness	[168]
CASC9	Up	MCF-7 cells	Doxorubicin	↑ Bcl-2	Apoptosis	[130]
lncRNA-ARA	Up	MCF-7 resistant to Adr	Adriamycin	↓ BAX	Apoptosis	[163]
PRLB	Up	human BC tissues and MDA-MB-231 cells	5-fluorouracil	↓ miR-4766-5p	Apoptosis	[153]
PTENP1	Down	Human BC tissues (*n* = 52) human BC cell lines MDA-MB-231, T-47D and MCF-7	Adriamycin	↑ PI3K/AKT ↓ Cleaved caspase 3 and PARP1	Apoptosis	[164]
HCP5	Up	MDA-MB-231, MDA-MB-157, MDA-MB468 cells	Cisplatin	↓ PTEN/↑ pAKT	Apoptosis	[165]
H19	Up	MDA-MB-453, MDA-MB-157, MDA-MB231 cells	Paclitaxel	↓ pAKT/BCL-2/Akt signaling pathway	Apoptosis	[166]
HOTAIR	Up	BC human tissues	-	miR-206/BCL-W	Apoptosis	[54]
LINC00628	Down	BC human tissues (*n* = 60), LCC2 and MCF-7 cells	-	BCL-2/BAX/Caspase-3	Apoptosis	[162]

## 7. Role of lncRNA in BC Chemoresistance through Regulation of Cancer Stemness

### 7.1. Stemness Regulation in Chemoresistant BC Cells

CSCs are sub-populations of cells that possess the ability to self-renew, differentiate, and undergo epithelial-to-mesenchymal transition (a key step in cancer metastasis) and are responsible for chemoresistance and poor prognosis in cancer treatments [4,169,170,171]. CSCs also have tumor-initiating capacity; however, they are different from tumor-initiating cells and possess distinctive properties such as efficient DNA repair, quiescence, and a strong influence on the tumor microenvironment, contributing to cancer progression, recurrence, and relapse [172,173]. CSCs have been documented in leukemia and many solid tumors, including BC [4,174,175]. In BC, a substantial increase in CSCs was noted in the residual tumors following exposure to conventional chemotherapy, suggesting that CSCs are resistant to treatment and associated with BC chemoresistance [4]. A higher percentage of CSCs was also detected in primary breast tumors following neoadjuvant chemotherapy confirming the initial result [176]. Also, in TNBCs, a positive correlation was observed between the expression of stem cell markers (CD44, ALDH1) and lower survival rates of TNBC patients, evidencing the importance of CSCs in TNBCs [177,178]. However, the mechanism behind CSC resistance is not well understood. Previous studies have shown that BCSCs overexpress various ABC transporters such as P-gp, ABCG2, ABCC1, and ABCB5 [20,179]. As mentioned above, these transporters can help BCs to pump out chemotherapeutic agents and reduce drug sensitivity [180,181]. HIF-1α is reported to be associated with early recurrence among women diagnosed with ER−BC [182]. Thus, its co-expression with CSCs may explain the poor prognosis and early recurrence, particularly in TNBC patients where BCSCs are highly expressed. In TNBC cells, a higher expression of ABC transporters, notably ABCG2, was also observed and was associated with a higher resistance to mitoxantrone [120]. Besides the efflux transporter, another mechanism of BC chemoresistance induced by CSCs is proposed. Ji and colleagues confirmed a series of BCSC surface biomarkers such as CD10, CD24, CD44, CD133, GPR77, ALDH1, EpCAM, and ABCG2, whose overexpression is an important cause of BCSC chemoresistance [20]. In a study by Su and colleagues, two cell-surface molecules, CD10 and GPR77, promoted tumor formation and chemoresistance by providing a survival niche for BCSCs [183]. In addition, a high CD44/CD24 ratio and ALDH1 were conserved during metastasis confirming the potential of these BCSC biomarkers in monitoring tumor progression and metastasis [184].

In conclusion, CSCs play a significant role in cancer progression, chemoresistance, and poor patient outcomes. Strategies to target and eliminate CSCs may hold the key to improving the effectiveness of cancer therapies and reducing the risk of recurrence. Further research into the mechanisms underlying CSC resistance is essential to develop more precise and effective treatments for cancer patients, particularly in cases like triple-negative breast cancer, where CSCs are highly expressed.

### 7.2. Regulation of Cancer Stemness by lncRNA in Chemoresistant BC Cells

Evidence shows the involvement of lncRNAs in the regulation of the cancer-related stemness (Table 7) [185,186,187]. In BCSCs, high expression of the novel lncROPM (a regulator of phospholipid metabolism) was observed and suggested to be required for the maintenance of BCSC properties both in vitro and in vivo. In clinic breast tumors and other solid tumors from BC patients, enhanced lncROPM was positively correlated with malignant grade/stage and poor prognosis. Mechanistically, lncROPM increased PLA2G16 expression and promoted the production of free fatty acids, especially arachidonic acid in BCSCs, thereby activating PI3K/AKT, Wnt/β-catenin, and Hippo/YAP signaling. Activation of these signaling pathways was thought to be involved in the maintenance of BCSC stemness [83]. The contribution of lncROPM and PLA2G16 to BCSCs chemoresistance was also suggested by the same study and is discussed in more detail in part II (regulation of lipid metabolism by lncRNA) [83]. The stemness properties can be influenced by lncRNAs, modulating the growth and apoptosis of cancer cells. Several studies have reported the mechanistic process of CSC regulation mediated by lncRNAs. Some lncRNAs regulate the acquisition of tumorigenic phenotypes through their association with quiescent SC populations. In MCF-7 cells, lncRNA-HAL was overexpressed in quiescent SCs, and its knockdown increased cell proliferation and impaired the proportion and function of CSCs, resulting in decreased tumor implantation in vivo [188]. LncRNA-LINC01133 was another lncRNA that stimulated the BCSC phenotype in triple-negative BC and acted as a malignancy indicator. LncRNA-LINC01133 was strongly induced by mesenchymal stem/stromal cells (MSCs) in BC cells and activated principal features of BCSC by targeting the miR-199a-FOXP2 axis [189]. Also, lncRNA-Hh induced BCSC properties. lncRNA-Hh can directly target GAS1, an enhancer of the Hedgehog signaling pathway, leading to the upregulation of Sox2 and Oct4 expression, and promoting the acquisition of stemness properties [190]. In TNBC, CD49f is known as a marker associated with chemoresistant and tumor-initiating cell populations [122]. It was identified that NEAT1 lncRNA expression was positively correlated with CD49f, suggesting a role for NEAT1 lncRNA in regulating chemoresistance of TNBC [191]. In another study, lncRNA NEAT1 was overexpressed in TNBC tissues and cell lines, and involved in cancer stemness and chemoresistance. The silencing of NEAT1 was able to decrease stem cell populations, such as ALDH+, CD44+/CD24−, and SOX2+, and increase the chemosensitivity of TNBC cells [168]. In this study, NEAT1 is highlighted as a significant contributor to breast cancer development, particularly in the TNBC subtype. It promotes cell proliferation, inhibits apoptosis, and plays a role in chemoresistance by modulating drug transporter genes and cancer stemness markers. These findings suggest that NEAT1 could be a potential therapeutic target in the treatment of breast cancer, especially in cases of chemoresistance.

In line with cancer chemoresistance, high expression of TUG1 lncRNA decreased temozolomide resistance of glioma cells by inhibiting the CSC-like properties. In fact, low expression of TUG1 lncRNA and high expression of Enhancer of zeste homolog 2 (EZH2) was observed in glioma cells. A172 glioma cells resistant to temozolomide also represent higher CSC-like properties compared to parental cells. The silencing of TUG1 or overexpression of EZH2 enhances CSC-like properties and resistance to temozolomide in A172 glioma cells [192]. EZH2 is a key epigenetic regulator that is suggested to be dysregulated in specific BC types, particularly in advanced stages [193,194]. Further studies on BC profiles are needed to understand how this important epigenetic regulator is altered and may lead to the discovery of the lncRNA regulator for EZH2. Fundamental studies on lncRNAs are also required to better understand mechanisms that contribute to BCSCs-associating chemoresistance. Overall, these findings underscore the critical role of lncRNAs in breast cancer stemness and chemoresistance. Targeting specific lncRNAs, such as NEAT1 and lncROPM, may hold promise as therapeutic strategies to combat breast cancer, especially in cases of chemoresistance. Further research is needed to unravel the complex interactions between lncRNAs and cancer stem cells, which could lead to the development of more effective treatments for breast cancer patients.

## 8. Role of lncRNA in BC Chemoresistance through Regulation of Autophagy

### 8.1. Regulation of Autophagy in Chemoresistant BC Cells

Autophagy is a homeostatic process that serves as a dynamic recycling system that produces new building blocks and energy for cellular renovation [195,196]. In cancer, autophagy plays a complex role. Studies show that autophagy can be activated following cytotoxic and metabolic stresses, such as hypoxia and nutrient deprivation. Its activation leads to cellular component recycling and confers stress tolerance, which serves to maintain tumor cell survival [197,198,199]. Elevated levels of autophagy have been observed in many cancers including breast tumors [200,201,202], suggesting a pro-oncogenic role for autophagy. It can also act as a tumor suppressor and play a role in cancer cell death [203,204,205,206]. The dual role of autophagy (tumor suppressor versus oncogenic) in BC progression is therefore evident [207]. Thus, autophagy is able to increase/decrease cellular viability, to activate/inhibit apoptosis, to induce/inhibit stemness, to promote/impair invasion and metastasis, and to increase/prevent drug resistance. In line with its tumorigenic role, studies showed that tamoxifen treatment induces autophagy-mediated resistance in MCF-7 and T-47D BC cells [208,209]. Also, in BC cells resistant to exemestane, activation of autophagy had a cytoprotective role in response to treatment. Interestingly, the inhibition of autophagy and/or the PI3K pathway reverted resistance by promoting apoptosis and the inhibition of cell survival pathways [210]. In TNBC cells resistant to PI3K/AKT inhibitors, treatment with ipatasertib and taselisib induces increased autophagy signaling and the inhibition of autophagy potentiates the therapeutic effect of PI3K/AKT inhibitors in combination with paclitaxel in TNBC models [202]. Also, in tamoxifen-resistant MCF-7 BC cells, the expression of Glucose transporter 1 (GLUT1) and autophagy flux were upregulated. The knockdown of GLUT1 sensitized cells to tamoxifen and significantly decreased autophagy flux in tamoxifen-resistant cell lines. Moreover, the inhibition of autophagy resulted in sensitization to tamoxifen [211]. A new mechanism of activation of autophagy was reported by Fedorova et al. in BC cells [212]. In this study, authors showed that Zeb1, the critical transcription factor of EMT, is involved in the regulation of autophagy in several BC cell models. Mechanistically, Zeb1 probably facilitates autophagy through the regulation of autophagic genes, resulting in increased LC3-II levels and increased resistance to genotoxic drugs in BC cells. In another study, higher expression of PLAC8 protein was correlated with ADR resistance in BC patients. This study proposed a suppressor role for autophagy in BC cells, regarding its dual role in cancer. In fact, in BC cells, PLAC8 inhibition decreased ADR resistance, while PLAC8 overexpression increased ADR resistance. Mechanistically, ectopic PLAC8 expression in MCF-7/ADR cells blocked the accumulation of the autophagy-associated protein LC3 and resulted in cellular accumulation of p62. Interestingly, autophagy activation significantly increased cellular response to ADR, while autophagy inhibition enhanced ADR resistance. This study suggests that PLAC8 inhibited autophagy through the participation of p62 and consequently resulted in ADR resistance in BC [206]. Further studies showed that in cancer cells and in conditions where mitochondria are targets of therapeutic molecules, activation of mitochondrial autophagy (mitophagy) can avoid the action of these molecules, leading to drug chemoresistance [213]. The flavonoid TL-2–8 has been shown to prevent BC by blocking mitophagy. Thus, TL-2–8, by blocking the fusion of the mitophagosome with the lysosome, improved the survival of mice bearing BC xenografts [214]. Fructose-1,6-bisphosphatase (FBP1), a gluconeogenesis rate-limiting enzyme, is generally considered to be a BC suppressor. FBP1 has been shown to induce apoptosis in human BC cells by inhibiting HIF-1α/BNIP3-mediated mitophagy [215]. Also, in BC cells resistant to DOX, the formation of mitophagolysosomes was induced in the presence of DOX treatment, thus proposing a role for mitochondrial autophagy in DOX resistance [58].

### 8.2. Regulation of Autophagy by lncRNA in Chemoresistant BC Cells

**Figure 4:**
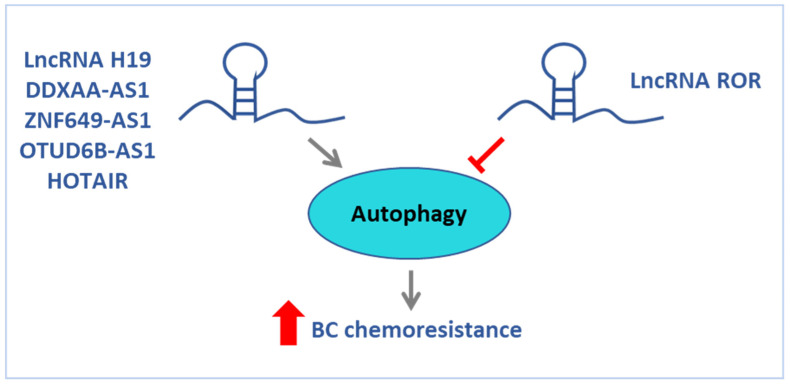
LncRNA players in regulation of autophagy associated with BC chemoresistance. LncRNAs can apply their tumorigenic role by either repressing tumor suppressor activity of autophagy (lncRNA ROR) or activating the tumorigenesis function of autophagy (H19, DDXAA-AS1, ZNF649-AS1, OTUD6B-AS1, and HOTAIR). 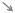 Shows activation and 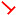 shows inhibition of autophagy. Red arrow shows increase in BC chemoresistance.

LncRNA-mediated autophagy may play an important role in mediating drug resistance in BC cells. Some lncRNAs apply their tumorigenic role by repressing autophagy activation while others have the opposite effect on the activity of autophagy (Figure 4 and Table 8). In a study by Li et al., lncRNA ROR (Regulator of Reprogramming) was found to have higher expression in both BC cells and tissues compared to normal cells and tissues. In this study, the authors also showed that in BC BT474 cells, transient inhibition of ROR increased the response to tamoxifen. In addition, transient inhibition of ROR increased *LC3 and Beclin 1* expression in the presence of tamoxifen. Interestingly, inhibition of autophagy reversed the effect of lncRNA ROR on drug resistance suggesting a tumor suppressor role for autophagy, which is mediated by the ROR lncRNA function [216]. Also, in a study by Wang et al. [217], they observed that the expression of H19 was substantially upregulated in a tamoxifen-resistant BC cell line and tumor tissues, and the knockdown of H19 enhanced the sensitivity to tamoxifen both in vitro and in vivo. Contrary to the ROR lncRNA, knockdown of H19 significantly inhibited autophagy in MCF7 tamoxifen-resistant (MCF7/TAMR) cells, while the overexpression of H19 promoted autophagy. Interestingly, the overexpression of H19 in MCF7 tamoxifen-sensitive cells induces tamoxifen resistance. Mechanistically, H19 regulates the autophagy-related gene *Beclin1* via epigenetic regulation and induces autophagy activation via the H19/SAHH/DNMT3B axis, contributing to tamoxifen resistance in BC. LncRNAs may affect the expression of Cis-trans ATG genes as well. In a study by Si et al. [218], DDX11-AS1 lncRNA was shown to be significantly upregulated in ADR chemoresistant BC cells and the overexpression of DDX11-AS1 promoted ADR resistance in BC. The interaction of DDXAA-AS1 with LIN28A was involved in DDX11-AS1-mediated ADR resistance as LIN28A increases the protein level of ATG7 and ATG12 by increasing their mRNA stability. Han and colleagues also showed that ZNF649-AS1 was more highly expressed in trastuzumab-resistant BC cells compared to sensitive cells. In the presence of trastuzumab treatment, ZNF649-AS1 was upregulated through epigenetic modification and knockdown of ZNF649-AS1 reversed trastuzumab resistance. Mechanically, ZNF649-AS1 was associated with the PTBP1 protein, which further promoted the transcription activity of the *ATG5* gene and autophagy activation [219]. Some lncRNAs regulate autophagy by binding to miRNAs as competitive endogenous RNAs (ceRNAs) to regulate miRNA expression, thus affecting autophagy. In TNBC cells resistant to paclitaxel, lncRNA OTUD6B-AS1 is identified to promote autophagy and DNA damage through the regulation of the miR-26a-5p/MTDH axis. Interestingly, the downregulation of OTUD6B-AS1 inhibited PTX-induced autophagy, while the upregulation of OTUD6B-AS1 and MTDH inhibited DNA damage response (DDR), promoted the autophagy process by the upregulation of LC3B-II and decreased PTX-mediated cell death [220]. Another mechanism for lncRNA-mediated autophagy is through the AKT/mTOR pathway, which is described for lncRNA-HOTAIR. Wang et al. found that HOTAIR can regulate oral squamous cell autophagy by regulating the mTOR pathway and inhibiting cell apoptosis and cisplatin sensitivity [221]. In BC, HOTAIR promotes resistance to tamoxifen and regulates the progression of BC through the activation of autophagy [222,223]. In a study by Li et al. [107], it was shown that inhibition of HOTAIR decreases DOX resistance in BC cells via the PI3K/AKT/mTOR signaling pathway.

In summary, it appears that many lncRNAs can contribute to BC chemoresistance through activating or inhibiting autophagy. Thus, the overexpression of H19, DDX11-AS1, OTUD6B-AS1, and HOTAIR induces drug resistance in BC cells by promoting autophagy. On the contrary, drug sensitivity is enhanced when autophagy is stimulated in response to the downregulation of ROR and ZNF649-AS1.

## 9. Role of lncRNA in BC Chemoresistance through Epigenetics Regulation

### 9.1. Epigenetic Regulation in Chemoresistant BC Cells

Epigenetics, the study of alterations in DNA methylation and modification of histones, has been shown to be associated with several types of cancer and especially with chemoresistance [224,225]. Histone modifications are correlated with several processes such as acetylation, deacetylation, methylation (hyper or hypomethylation), phosphorylation, and ubiquitination [226,227,228]. DNA methylation and histone acetylation are deemed the most significant alterations to trigger BC resistance. For instance, hypermethylation of ESR1 culminated in reduced ERα expression among approximately 20% of the patients administrated with tamoxifen [229]. Furthermore, HATs catalyze the acetylation of histones, TFs, and heat shock proteins, leading to resistance in patients administered tamoxifen [230]. Thereafter, the role of these epigenetic modifications in the development of BC chemoresistance is discussed.

DNA methylation: The methylation of DNA is the process by which methyl groups are reversibly added to the fifth carbon position of the cytosine of S-adenosylmethionine (SAM) by DNA methyltransferases (DNMTs)—DNMT1, DNMT3A, and DNMT3B [231]. Irregular DNA methylation can occur by hypermethylation in the promoter regions of tumor suppressor genes, which leads to their silencing, while hypomethylation in the promoter regions of oncogenes can activate them [232]. The influence of DNA methylation in some genes interferes with cell differentiation, DNA binding, homeobox proteins, and transcriptional signaling. As well, DNA methylation also affects some processes involved in the growth of neoplastic cells, including chromatin remodeling, transcriptional control, DNA repair, cell cycle control, apoptosis, and metabolism [233]. Changes in DNA methylation lead to altered cell adhesion, tissue invasion, and metastasis pathway genes [234]. DNA methylation influences the tumor microenvironment, leading to changes in tumorigenesis–metastasis properties [235] and chemoresistance [232,236]. The hypomethylation seen in the epigenetic profile of BC cells differs from normal breast cells. Aberrant changes in DNA methylation are frequent events during BC progression and chemoresistance acquisition. Despite being heterogeneous among BC patients, hypomethylation in the genes *ALDH1L1*, *HOPX*, *WNT5A*, and *SOX9* was identified and associated with stem cell quiescence after obtaining chemoresistance [237]. Interestingly, overexpression of hypomethylated miR-663 induces chemoresistance to cyclophosphamide and docetaxel in BC cells by downregulating heparin sulfate proteoglycan 2 (HSPG2) [238]. Moreover, the hypomethylation of the ABCB1 promoter results in the upregulation of the ABCB1 protein and the acquisition of resistance to taxanes through efficient drug efflux [239]. Furthermore, in patients with ER-positive breast cancer chemoresistant to tamoxifen, hypomethylation of DNA binding inhibitor 4 (ID4) was observed [240]. Another mechanism for acquiring resistance to drug therapy is through hypermethylation. According to Palomeras et al. [241], epigenetic silencing by hypermethylation of *TGBI* (Transforming Growth Factor Beta Induced) is associated with trastuzumab resistance in HER2+ BC patients. In addition, Zhang et al. [242], in 2017, studied the formation of the ZEB1/DNA methyltransferase (DNMT)3B/histone deacetylase (HDAC)1 complex on the ER-α promoter, which leads to DNA hypermethylation and the silencing of ER-α. Thus, ZEB1 represses ER-α transcription and confers antiestrogen resistance in BC.

Histone acetylation: As one of the most common epigenetic modifications, histone acetylation can neutralize lysine’s positive charge to relax the chromatin structure and enhance transcriptional activity [226]. MCF-7 and MDA-MB-231 BC cell lines resistant to DOX showed epigenetic aberrations associated with the acquisition of resistance to this drug. These cells show a significant increase in H3 acetylation and methylation, as well as hypermethylation-mediated inactivation of the *MSH2* gene promoter, which has been associated with the acquired resistant phenotype [243]. Also, the P300/CBP family mediates alterations in the histone acetylation landscape, promoting the relaxation of chromatin, and allowing the binding of transcriptional factors to activate transcription [244]. P300/CBP contributes to the transcription of oncogenes and tumor suppressor genes (*TSGs*), which promote or inhibit numerous BC-related processes, among them apoptosis [245] and drug resistance [246,247]. It was also observed that histone H3 and H4 acetylation in the MDR1 promoter leads to the overexpression of MDR1 in BC, one of the main causes of chemoresistance [248]. Finally, Nayak et al. demonstrated that histone variants H2A and H2B are mediators of drug resistance and sensitivity in breast cancer [249].

### 9.2. Regulation of Epigenetic Control by lncRNA in Chemoresistant BC Cells

**Figure 5:**
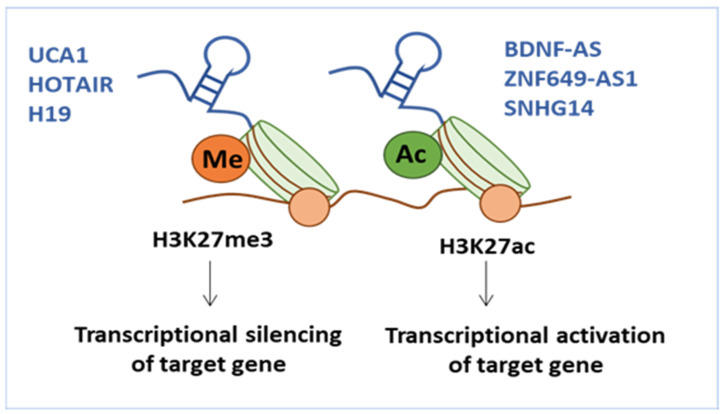
LncRNA players in epigenetic regulation associated with BC chemoresistance. LncRNAs can control epigenetic regulation through both histone methylation and acetylation, leading to transcriptional silencing or activation of the target gene, respectively.

One of the main mechanisms of action of lncRNAs is through epigenetic regulation, which mainly includes DNA methylation and histone modifications (Figure 5 and Table 9). LncRNA UCA1, an oncogene in BC, can induce tamoxifen resistance via inhibiting the mTOR signaling pathway [250]. Li and colleagues, in 2019, suggested that the molecular mechanism involved in UCA1-induced tamoxifen resistance was physically associated with zeste homolog enhancer 2 (EZH2), which suppressed *p21* expression through histone methylation (H3K27me3) in the *p21* promoter. So, in this way, UCA1 regulates the EZH2/*p21* axis and the PI3K/AKT signaling pathway in BC in tamoxifen resistance [250]. Another lncRNA has been positively correlated with tamoxifen resistance, the HOTAIR. This lncRNA conducts the recruitment of a histone methyltransferase EZH2, epigenetically represses gene expression, and promotes BC progression. Zhuang et al. [251], in 2015, demonstrated that with a variant of MCF-7, MCF-7-TNR, cells exhibited an increase in HOTAIR expression after surviving progressive exposure to tumor necrosis factor-α (TNF-α) and various chemotherapeutic agents. LncRNA HOTAIR enhances ER signaling and confers tamoxifen resistance in BC. Mechanistically, HOTAIR is a direct target of ER-mediated transcriptional repression and is thus restored upon the blockade of ER signaling, either by hormone deprivation or tamoxifen treatment [222].

BDNF-AS lncRNA induces tamoxifen resistance and malignant progression of endocrine-resistant and triple-negative BC. BDNF-AS exhibited the highest H3K27ac expression and signals in MCF-7R compared to MCF-7 cells. Higher levels of this histone acetylation (H3K27ac) in tamoxifen-resistant cells compared to tamoxifen-sensitive cells suggest an epigenetic activation. The epigenetic regulation of BDNF-AS is to activate the mTOR pathway, promoting the degradation of the RNH1 protein (a ribonuclease inhibitor), through TRIM21-mediated ubiquitination (E3 ubiquitin-protein ligase whose activity is dependent on E2 enzymes) from RNH1 to K225. BDNF-AS lncRNA suppresses RNH1-regulated and RISC-mediated (an RNA-induced silencing complex) mTOR mRNA downregulation, thus maintaining activation of mTOR signaling. Importantly, mTOR inhibitors, but not PI3K inhibitors, can reverse tamoxifen resistance induced by BDNF-AS overexpression [252]. Han et al. [219], in 2020, demonstrated that the lncRNA ZNF649-AS1 induces trastuzumab resistance by acetylation of lysine 27 on histone H3 protein, i.e., modifying H3K27ac via association with polypyrimidine tract binding protein 1 (PTBP1), which promotes autophagy through *ATG5* expression.

Chromatin immunoprecipitation (ChIP) assays demonstrated that the lncRNA SNHG14 can induce PABPC1 expression through modulation of the acetylation of H3K27 in the *PABPC1* gene promoter, resulting in the activation of the Nrf2 signaling pathway. LncRNA SNHG14 contributed to trastuzumab tumorigenesis and resistance in BC through the regulation of *PABPC1* expression by H3K27 acetylation [253]. Dong and colleagues, in 2018, demonstrated that the long noncoding RNA SNHG14 induces BC trastuzumab resistance by regulating the expression of *PABPC1*, which is activated via the Nrf2 signaling pathway, through the acetylation of H3K27 [253]. High expression of lncRNA H19 was identified as a powerful factor associated with paclitaxel resistance in ERα-positive BC cells through epigenetic silencing of the pro-apoptotic gene *BIK*, through the recruitment of EZH2 and trimethylation of histone H3 to lysine 27 [254]. Basak et al. proved that lncRNA H19 acts as an estrogen receptor modulator required for resistance to endocrine therapy in ER+ BC cells [166,255]. Therefore, epigenetic changes lead to drug resistance and can also transfer information through extracellular vesicles (EVs). A review by Ashekyan et al. [256] showed that biomolecules, such as miRNAs, lncRNAs, and the recently discovered circular RNA, are being secreted from BC cells or BC tumor microenvironment cells from EV. As exemplified by Wang et al., in 2020, the EV-mediated transfer of H19 RNA induces DOX resistance in breast cancer [47]. In addition, Tang et al. [257] showed serum exosomal HOTAIR overexpression correlated with poor survival and poor response to chemotherapy in patients with breast cancer.

In conclusion, the involvement of lncRNAs in BC drug resistance is intricately linked to their ability to exert epigenetic regulation through DNA methylation and histone modifications. These lncRNAs can influence drug resistance by interacting with histone-modifying enzymes, such as EZH2, or by affecting histone acetylation (H3K27ac), ultimately impacting the expression of genes involved in drug response and tumor progression. Additionally, lncRNAs can be transferred via extracellular vesicles (EVs), allowing them to mediate drug resistance and communicate vital information within the breast cancer microenvironment. Understanding the epigenetic regulation mediated by lncRNAs provides valuable insights into the molecular mechanisms underlying drug resistance in breast cancer. Targeting these epigenetic interactions may open up new avenues for therapeutic interventions, offering hope for more effective treatment strategies and improved outcomes for breast cancer patients.

## 10. Concluding Remarks

LncRNAs are a class of transcripts more than 200 nucleotides in length and with limited protein-coding ability, which have been shown to function as master regulators for gene expression. LncRNAs regulate gene expression through a complex process involving multiple mechanisms that have not yet been fully understood [258]. They can regulate gene expression at the transcriptional level by binding to DNA directly or indirectly by binding to transcription factors. They can also regulate gene expression at the post-transcriptional level by targeting mRNAs, miRNAs, or proteins and modulating their activities and stability. Finally, lncRNAs can control epigenetic regulation by interfering with chromatin complexes [259]. Such mechanisms allow lncRNAs to play a critical role in many cellular and genomic processes associated with carcinogenesis and drug resistance/sensitivity. LncRNAs are involved in the main mechanisms associated with BC chemoresistance such as metabolic reprogramming [68,69,71,72,83,84,99,100,101,102,103,104,105,106,260,261,262,263], altered drug efflux [125,129,130,264,265], maintaining cancer stemness [185,186,187], and epigenetic regulation [201,228,233,234]. Thus, the understanding of these mechanisms in deep detail may interest the clinical outcomes of BC patients and can be used as therapeutic targets to overcome BC therapy resistance.

Studies have shown an increased drug response following lncRNA targeting in BC preclinical models [131,266]. For example, the targeting of FTH1P3 lncRNA by shRNA suppressed the tumor growth of paclitaxel-resistant BC cells in a xenograft mice assay [119]. Also, targeting LINK-A lncRNA has been seen in a preclinical study to improve the BC sensitivity to immune checkpoint inhibitors [266]. Conversely, some lncRNAs improve therapeutic response, thus suggesting a dual role for lncRNAs in drug chemoresistance. For instance, lncRNA GAS5 has been found to have a suppressive role in MCF-7/ADR BC cell lines and GAS5 overexpression significantly enhanced the ADR sensitivity to ADR treatment [129]. Therefore, a combination therapy consisting of both chemotherapeutic drugs and suppressor lncRNAs provides a promising strategy to control BC chemotherapy resistance.

In this review, we show evidence of several lncRNAs interfering with metabolism regulation, efflux transporters activity, control of apoptosis and autophagy, cancer stemness, and epigenetic regulation via different pathways causing chemoresistance in BC. Considering the deep alteration of their expression profile in cancer cells, lncRNAs could not only be considered as potential biomarkers for early diagnosis of cancer patients but can also be useful to monitor cancer progression and, finally, to evaluate chemoresistance. Some lncRNAs are already approved by the FDA for diagnostic biomarkers, which is the case for PCA3 lncRNA in prostate cancer. LncRNA H19 is also at the stage of clinical trials for glioblastoma, ovarian, bladder, and pancreatic cancer [267]. For breast cancer, a recent work of Pourramezan et al. [268] established a list of fifteen circulating lncRNAs that are differentially expressed in BC patients compared to healthy women. They evidenced that the downregulation of the lncRNA NKILA and NBAT1 are linked to tumor size, whereas the blood concentration of H19 and SPRY4-IT1 is a good parameter to discriminate between BC patients and controls. Further studies are needed to confirm these observations in other BC models.

From a perspective of the development of more personalized therapy, the expression profile of lncRNA could be useful to predict cancer cell sensitivity to different chemotherapeutic agents and thereby decreasing the chemoresistance. Moreover, identifying the lncRNAs that are expressed only in chemoresistant cells could offer new therapeutic alternatives to bypass chemoresistance and cancer recurrence. The lncRNAs listed in this review could represent interesting targets to inhibit the intracellular mechanisms that are involved in the chemoresistance of BC cells.

## Figures and Tables

**Table 1:** The four main archetypes of lncRNA mechanisms of action.

Archetype	Intracellular Compartment	Mechanism of Action
GUIDE	Nucleus	Recruitment of target protein complexes (ribonucleoprotein complexes, transcription factors, or chromatin-modifying enzymes) in gene promoters to activate transcription
SCAFFOLD	Nucleus	Help in the assembly of nucleoriboprotein complexes
DECOY	Nucleus/Cytosol	Binding with regulatory proteins to repress DNA transcription and with miRNA to prevent their binding to their intrinsic targets
SIGNALING	Nucleus	Regulation of the transcription of target genes alone or by interacting with chromatin-modifying enzymes to inhibit gene transcription

**Table 2:** Summary of studies that reported the role of lncRNAs in regulation of BC tumorigenesis and chemoresistance by modulating glucose metabolism. ↑ shows positive regulation and ↓ show negative regulation of target protein or miRNA.

LncRNA	Expression Pattern	Human/Animal/ Cell Lines	Treatment	Possible Pathway	Reference
DIO3OS	Up	Human tumor samples (*n* = 257)	Aromatase inhibitor	PTBP1/LDHA ↑ LDHA expression	[68]
ANRIL	Up	TNBC cells (MDA-MB-231 and BT-549 cells)	Doxorubicin	↓ miR-125a/↑ enolase 1	[69]
YIYA	-	MCF-7 and MD-MBA-45 BC cells	-	CDK6/↑ PFKFB3	[70]
SNHG3	Up	MD-MBA-45 BC cells	-	↓ miR-330-5p/↑ PKM2	[71]
SNHG7	Up	MDA-MMB-436, HS578T, SKBR3, MDA-MB-231, and MCF-7 BC cells/Human tumor samples (*n* = 30)	Doxorubicin, Paclitaxel	↓ miR-34a/↑ LDHA	[72]

**Table 3:** Summary of studies that reported the role of lncRNAs in regulation of BC tumorigenesis and chemoresistance by modulating lipid metabolism. ↑ shows positive regulation of target protein or miRNA.

LncRNA	Expression Pattern	Human/ Animal/ Cell Lines	Treatment	Possible Pathway	Reference
LncROPM	Up	BT-549, Hs578T, MDAMB-231, MDA-MB-468, MDA-MB-436, MCF-7, and T47D cells	Doxorubicin, Cisplatin, Tamoxifen	↑ *PLA2G16*/PI3K/AKT, Wnt/β-catenin, Hippo/YAP	[83]
LINC00467	Up	MCF-7 and MDA-MB-231	Deoxycytidine	↑ miRNAs/*TGFB2*/TGF-β	[84]
OLMALINC	Up	MDA-MB-231 and 8T149 cells	-	↑ SCD1 expression	[85,86]

**Table 7:** Summary of studies that reported the role of lncRNAs in regulation of BC tumorigenesis and chemoresistance by modulating cancer stemness.

LncRNA	Expression Pattern	Human/Animal/ Cell Lines	Treatment	Possible Pathway	Dysregulated Mechanism	Reference
lncROPM	Up	BT-549, Hs578T, MDAMB-231, MDA-MB-468, MDA-MB-436, MCF-7, and T47D cells	Doxorubicin, Cisplatin, Tamoxifen	PI3K/AKT, Wnt/β-catenin, and Hippo/YAP	Cancer stemness	[183]
LncRNA-HAL	Up	Human BC tissues (42 ER+ and 42 TNBC), MCF-7 cells	-	Quiescent stem cell population	Cancer stemness	[188]
LINC01133	Up	MDA-MB-231, MDA-MB-468	-	KLF4/miR-199a/ FOXP2	Cancer stemness	[189]
lncRNA-Hh	Up	MCF-7, Hs578T, BT549, MDA-MB-231 cells	-	Hedgehog signaling pathway	Cancer stemness	[190]
NEAT1	Up	BC patients (179 patients and 192 control), In vivo xenograft animal model, MDA-MB-231 cells	Cisplatin, Taxol	-	Cancer stemness	[168,191]

**Table 8:** Summary of studies that reported the role of lncRNAs in regulation of BC chemoresistance by modulating autophagy. ↑ shows positive regulation and ↓ show negative regulation of target protein or miRNA.

LncRNA	Expression Pattern	Human/Animal/ Cell Lines	Treatment	Possible Pathway	Dysregulated Mechanism	Reference
ROR	Up	Human BC BT474 cells and tissues	Tamoxifen	*LC3* and *beclin 1*	Autophagy	[216]
H19	Up	MCF-7 cells	Tamoxifen	↑ SAHH/DNMT3B	Autophagy	[217]
DDX11-AS1	Up	Human BC ZR-751 and MCF-7 cells	Adriamycin	↑ LIN28A/ATG7 and ATG12	Autophagy	[218]
ZNF649-AS1	Up	Human BC tissues (*n* = 90) and SKBR-3 and BT474 cells	Trastuzumab	↑ PTBP1/*ATG5*	Autophagy	[219]
OTUD6B-AS1	Up	Human BC MDA-MB-231 and HCC1937 cells	Paclitaxel	↓ miR-26a-5p/↑ MTDH ↑ LC3B-II	Autophagy	[220]
HOTAIR	Up	Human BC MCF-7 and SKBR3 cells	Doxorubicin	PI3K/AKT/mTOR	Autophagy	[221,222]

**Table 9:** Summary of studies that reported the role of lncRNAs in regulation of BC chemoresistance by modulating epigenetic regulation. ↑ shows positive regulation and ↓ show negative regulation of target protein or miRNA.

LncRNA	Expression Pattern	Human/Animal/ Cell Lines	Treatment	Possible Pathway	Dysregulated Mechanism	Reference
UCA1	Up	MCF-7; T-47D; LCC2; LCC9	tamoxifen	↑ EZH2/↓ *p21* axis; ↑ PI3K/AKT pathway	Epigenetic control	[250]
HOTAIR	Up	MCF-7; T-47D	tamoxifen; TNF-a	↑ ER signaling; ↑ SRC and p38MAPK kinases; ↑ EZH2	Epigenetic control	[222]
BDNF-AS	Up	MCF-7; T-47D; MDA-MB-231	tamoxifen	↑ RNH1/TRIM21/mTOR	Epigenetic control	[252]
ZNF649-AS1	Up	SK-BR-3; BT474	trastuzumab	↑ *ATG5* through associating with PTBP1	Epigenetic control	[219]
SNHG14	Up	SKBR-3; BT474	trastuzumab	↑ *PABPC1*; ↑ Nrf2 pathway	Epigenetic control	[253]
H19	Up	ZR-75-1; MCF-7	paclitaxel	↓ *BIK*; ↓ NOXA	Epigenetic control	[254]

## Abbreviations

ADR	Adriamycin
ANRIL	Antisense noncoding RNA in the INK4 locus
ARA	ADR resistance associated
ATP	Adenosine triphosphate
BAX	Bcl-2-Associated X
BC	Breast cancer
BCRP	BC resistance protein
Bcl-xl	B-cell lymphoma extra-large
BH3	Bcl-2 homology 3
CAF	Cancer-associated fibroblasts
CAM-DR	Cell-adhesion-mediated drug resistance
CASC9	Cancer susceptibility candidate 9
CDK6	Cyclin-dependent kinase 6
CeRNAs	competitive endogenous RNAs
ChiP	Chromatin immunoprecipitation
COL1	Collagen type 1
CSC	Cancer stem cells
DDR	DNA damage response
DKK2	Dickkopf-related protein 2
DNA	Deoxyribonucleic Acid
DNMTs	DNA methyltransferases
DOX	Doxorubicin
DRP1	Dynamin-related Protein 1
EIF5A2	Eukaryotic Translation Initiation Factor 5A2
EMT	Epithelial–mesenchymal transition
EV	Extracellular vesicle
EZH2	Enhancer of zeste homolog 2
FAO	Fatty acid oxidation
FASN	FA synthase
FGFR4	Fibroblast Growth Factor Receptor 4
FTH1P3	Ferritin heavy chain 1 pseudogene 3
GLUT1	Glucose transporter 1
HCP5	Histocompatibility leukocyte antigen complex P5
HDAC	Histone deacetylase
HIF	Hypoxia Inducible Factor
HK2	Hexokinase 2
HOTAIR	HOX antisense intergenic RNA
ITGB1	β1-Integrin
LATS1	Large tumor suppressor kinase 1
LDHA	Lactate dehydrogenase A
LINK-A	Long intergenic noncoding RNA for kinase activation
LINP1	LncRNA In Non-Homologous End Joining Pathway 1
LncRNA	Long noncoding RNA
OLMALINC	oligodendrocyte maturation-associated long intervening noncoding RNA
lncRNA-PRLB	Progression-associated lncRNA in BC
LncROPM	Regulator of phospholipid metabolism
MALAT-1	Metastasis-associated lung adenocarcinoma transcript 1
MAPK	Mitogen-activated protein kinase
Mdivi-1	Mitochondrial Division Inhibitor 1
MDM2	Mouse double minute 2
MDR	Multidrug resistance
MRP1	Multidrug-resistant protein-1
mtDNA	Mitochondrial DNA
MTFR2	Mitochondrial Fission Regulator 2
MTLN	Mitoregulin
NEAT1	Nuclear-enriched abundant transcript 1
OXPHOS	Oxidative phosphorylation
PA	Phosphatidic acids
PARP1	Poly (ADP-ribose) polymerase1
PCD	Programmed cell death
Pep1	Pro-fusion cell-permeant peptide
PFK1	Phosphofructokinase 1
PFKFB3	6-phosphofructo-2-kinase/fructose 2,6-bisphosphatase 3
P-gp	P-glycoprotein 1
PIM2	Proviral Insertion in Murine Lymphomas 2
PKM2	Pyruvate kinase musculaire 2
PLA2	Phospholipase A2
PLAC8	Placenta Associated 8
POU3F3	Pou class 3 homeobox 3
PTBP1	Polypyrimidine tract binding protein 1
PTEN	Phosphatase and tensin homolog
PTENP1	PTEN pseudogene 1
PTX	Paclitaxel
RAC1	Ras-related C3 botulinum toxin substrate 1
RACGAP1P	Pseudogene of Rac GTPase activating protein 1
ROR	Regulator of Reprogramming
ROR1	Receptor Orphan tyrosine kinase-like Receptor 1
ROS	Reactive oxygen species
SAM	S-adenosylmethionine
SncmtRNA	Sense mitochondrial ncRNA
SNHG	Small Nucleolar RNA Host genes family
TGFB2	Transforming growth factor beta 2
TNBC	Triple-negative breast cancer
TNF-α	Tumor necrosis factor-α
TRPS1	Transcriptional Repressor GATA Binding 1
TUG1	Taurine-upregulated gene 1
XIST	X inactive-specific transcript
